# Kidney Biopsy in a Pregnant Patient with Suspected Glomerular Disease: Commentary

**DOI:** 10.34067/KID.0000000000000244

**Published:** 2023-10-26

**Authors:** Michelle A. Hladunewich

**Affiliations:** Division on Nephrology, Department of Medicine, Sunnybrook Health Sciences Centre, Temerty Faculty of Medicine, University of Toronto, Toronto, Ontario, Canada

**Keywords:** ANCA, GN, lupus nephritis, membranous nephropathy

I read with interest the arguments for and against kidney biopsy in pregnancy, as by in large, both pairs of authors arrived at the same general consensus statement. *A kidney biopsy in pregnancy can occur so long as the information gleaned outweighs the risk of the procedure.* This balance between benefit and risk though must be assessed on many levels and varies by gestational age, practice setting, availability of alternate diagnostic tests, and treatment options (Table 1). As such, I will present an approach that each clinician can consider when managing these challenging cases. Both groups of authors made excellent points, but ultimately, the decision must be individualized.

**Table 1:** Factors to consider before a kidney biopsy in pregnancy—biopsy checklist

Gestational Age	Early safer than late • Progressive anatomical challenges due to an expanding uterus • Superimposed preeclampsia becomes more likely at pregnancy progresses
Procedural skill	Centers experienced with biopsy in pregnancy
Other safety factors	BMI, hemoglobin, platelet count, coagulation parameters or imminent need for anticoagulation
Available alternative diagnostic tests	Urinalysis GN screen • Serology for SLE, ANCA-associated vasculitis, anti-GBM disease • Anti-PLA2R antibody for membranous nephropathy • Genetic testing for hereditary nephritis Preeclampsia diagnostic tools • Dropping fetal growth percentile • Placental Doppler examination (PI >1.4) • Angiogenic factors (increased soluble Flt-1/PlGF ratio or just depressed PlGF)
Available treatment options	Pregnancy safe immunosuppression includes prednisone, calcineurin inhibitors, azathioprine, eculizumab, and rituximab if used early in gestation
Patient perspectives	Will information from a biopsy guide decision making with respect to termination versus continuation of pregnancy (*e.g.*, acuity versus chronicity) Patient tolerance for loss of kidney function Patient desire to continue pregnancy irrespective of heightened renal risk

BMI, body mass index; GBM, glomerular basement membrane; PLA2R, phospholipase A2 receptors; PI, pulsatility index; Flt-1, fms-related receptor tyrosine kinase 1; PlGF, placental growth factor.

Kidney biopsy remains the gold standard for diagnosis and provides important prognostic information; and at least in the nonpregnant population, kidney biopsy has become a relatively low-risk procedure.^1^ So perhaps the first question to consider is the safety of the procedure at your institution, most importantly institutional experience and comfort performing this typically rare procedure in a pregnant woman. Certainly, earlier in gestation is better given the progressive anatomical challenges due to an expanding uterus, but one must also add to this risk assessment the other usual factors, including body habitus, renal size, BP control, hemoglobin, platelet count, and coagulation parameters because all can affect the safety of the procedure. Contrary to Oliveira and Hendren's Prostatement noting that the need for anticoagulation might push one to biopsy, a high likelihood for imminent thromboembolism and the need for anticoagulation should in my opinion inform against biopsy (*e.g.*, a serum phospholipase A2 receptor–positive severely nephrotic patient).

This careful, thoughtful approach is perhaps best illustrated in a recent publication from Mexico City.^2^ They reviewed the safety of 20 biopsies in pregnancy conducted between 2015 and 2019. Each case first had a multidisciplinary review to help deliberate the need for the procedure and how it might guide treatment. They reported five minor complications (hematoma and/or hematuria) and only a single significant psoas bleed, requiring transfusion. Of note, this is a very experienced center that conducts many biopsies yearly (≈250 biopsies per year—personal communication from authors) with the vast majority in this pregnancy series requiring only two passes for adequate tissue.

Once convinced that biopsy is indeed safe, the availability of alternate diagnostic investigations and available treatment options should then be considered. Serology for lupus nephritis or vasculitis, for example, can confirm a diagnosis in those with systemic illnesses, while phospholipase A2 receptor for the diagnosis of membranous nephropathy is becoming more widely available. Furthermore, if preeclampsia has entered into the differential diagnosis, biopsy is precarious and not recommended. The growth trajectory of the baby, placental Doppler results, and, where available, antiangiogenic factors can all aid with the diagnosis of superimposed preeclampsia, which often overlays the underlying renal disease as pregnancy progresses. Again, speaking to the rationale for favoring the procedure earlier in gestation before superimposed preeclampsia has potentially complicated the presentation and increased the precariousness of the procedure.

Available treatment options also factor into the need for more information. Although the conclusion from the aforementioned biopsy study was that information-guided therapy, every single patient received prednisone and tacrolimus with or without azathioprine.^2^ Similarly, in a study that looked at the value of biopsy in patients with lupus nephritis, the most typical treatment change was escalation of steroid dosing and/or the addition of hydroxychloroquine and azathioprine.^3^ In one case, cyclosporin was added, and in two pregnancies, it was said the biopsy facilitated the decision to terminate.^3^ I would argue that in many of these cases the same treatment decisions could have been made in the context of clinical care even in the absence of biopsy data. Every pregnant patient with systemic lupus should be on hydroxychloroquine. Significant worsening of kidney function or proteinuria early in pregnancy in a woman with established lupus nephritis, vasculitis, or other difficult-to-manage glomerular diseases always warrants a discussion about termination as an option irrespective of biopsy findings. Otherwise, treatment options are limited to a handful of safe drugs that we typically use in combination^4^ because even outside pregnancy, there are data to support multitargeted therapy in glomerular disease.^5^

Finally, the most important decision aid at your disposal are the many discussions with mom and careful shared decision making. Pregnancy counseling is not for the faint of heart. It requires a significant investment of time to build a trusting doctor–patient relationship and to understand a woman's personal circumstances and how a child will affect her and her family. Many women with kidney disease are diagnosed during pregnancy because this is their first health care encounter. So a diagnosis in some cases previability may help guide decision making. Acuity versus chronicity may guide next steps especially in resource-constrained environments where dialysis is either unavailable or costly. Furthermore, loss of kidney function during a pregnancy in some situations may prove to be too significant a personal cost. While others may choose to carry a pregnancy despite significant renal risks.

The complexity of shared decision making is likely best illustrated by real clinical scenarios. Some years ago, we published a case of a woman who presented at 27 weeks of gestation with nephrotic syndrome accompanied by severe oligohydramnios, but normal placental Dopplers and fetal growth, ruling out preeclampsia.^6^ All serological studies were unremarkable, so a biopsy was conducted at 30 weeks of gestation, diagnosing focal segmental glomerulosclerosis. Both the nephrotic syndrome and oligohydramnios improved with prednisone therapy, and the biopsy did guide diagnosis and therapy. She delivered a healthy baby at 37 weeks after a spontaneous birth. I contrast this to another case of a woman with known systemic lupus and nephritis who became pregnant after self-discontinuation of mycophenolate mofetil. With her creatinine peaking at 4.95 mg/dl along with 5 g of proteinuria, I did not need a confirmatory biopsy to tell me she had aggressive lupus nephritis likely on the background of significant chronicity. During counseling, it became clear that no amount of additional information that I could provide her would convince her to terminate her pregnancy nor would she agree to exposure of a potential teratogen, so biopsy was not pursued as that would simply add risk without altering management options. She was treated aggressively with pulse methylprednisolone, then high-dose oral prednisone as well as azathioprine during pregnancy and converted promptly to mycophenolate mofetil at 6 weeks *postpartum*. Although her renal parameter improved, she developed superimposed preeclampsia and delivered her baby at 27 weeks weighing 780 g. Both mom and baby, fortunately, are well.

In summary, the approach to biopsy in pregnancy was best articulated by Day and colleagues in 2008 and in my opinion has not changed appreciably.^7^ They note that biopsy during pregnancy can provide valuable information in a carefully selected subgroup of women.

As opposed to arbitrarily setting gestational ages and/or conditions wherein biopsy is permissive, every woman instead requires and deserves an individualized thoughtful approach wherein the risk of a biopsy is weighed against the potential for gaining of new information that will alter the course of treatment and more importantly assist her with critical decision making.
